# Corn or Climate? Climate Change Facilitated, but Did Not Initiate, the Northward Range Expansion of the Green Jay in Texas

**DOI:** 10.1002/ece3.74513

**Published:** 2026-10-18

**Authors:** Callen M. Inman, Lawrence E. Gilbert

**Affiliations:** ^1^ Brackenridge Field Laboratory The University of Texas Austin Texas USA; ^2^ Department of Integrative Biology The University of Texas at Austin Austin Texas USA; ^3^ Department of Biological Sciences Louisiana State University Baton Rouge Louisiana USA

**Keywords:** anthropocene, climate change, landscape ecology, ornithology, wildlife management

## Abstract

Anthropogenic changes are driving global shifts in species distributions. Two highly divergent bird species, the green jay ( *Cyanocorax yncas* ) and the blue jay ( *Cyanocitta cristata* ), recently hybridized in Texas following range expansions. While the southwestern spread of the blue jay has been attributed to suburban bird‐feeding, the cause of the green jay's > 2° northward expansion over the last 70 years is less clear. Climate change has been proposed as a driver, but concurrent changes in habitat and resource availability—including widespread supplemental feeding of deer—complicate interpretation. We combine ornithological records, eBird observations, climate data, and economic data to investigate the progression of the green jay's range expansion in relation to climate and supplemental feeding. The first records of resident green jays north of southernmost Texas occurred in the 1960s. Sustained northward expansion was detectable from 1980 to 2021 and coincided with reduced severe freeze frequency from 1982 to 1996. Severe winters occurred alongside a reduced probability of green jay detections in newly occupied locations. However, range expansion increased during the period with the coldest average winter in the 95‐year climate record and coincided with growing supplemental feeding. We suggest that winter food supply on widespread hunting ranches and habitat loss drove initial northward colonization by green jays. Subsequent warming may have then relaxed climatic constraints once this novel resource became widespread. This potential interaction between novel resources and climate change highlights that different anthropogenic mechanisms may work together to alter species distributions.

## Graphical abstract

The green jay's range expansion in Texas challenges preconceived notions about how anthropogenic change is affecting species distributions. We show that while the green jay's range expansion coincided with a period of warming and may have increased in response to warmer winters, this process began during the period of the 20th Century with the coldest winters. 70 years of natural history observations in South Texas suggest that supplemental feeding of deer may have played a role in driving the northward expansion of this tropical bird in spite of unfavorable climatic conditions.

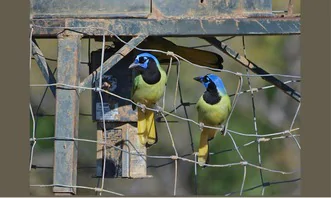

## 1 Introduction

In an era of global anthropogenic change, the distributions of a broad range of taxonomic groups are shifting (Boivin et al. 2016; Barton et al. 2016). Such distributional shifts often occur in ways that track changes in climate, such as in poleward (Hickling et al. 2006) or upslope elevational shifts (Schai‐Braun et al. 2021). However, simultaneous anthropogenic changes in habitat and resource availability may trigger range shifts that augment, negate, or obscure the effects of climate change (Lenoir et al. 2010; Tingley et al. 2012; Rubenstein et al. 2023). In general, multiple dimensions of anthropogenic change warrant consideration when understanding the drivers of range shifts (Morelli et al. 2025).

Isolating climate change from other potential drivers of range shifts is not trivial, especially in regions of the world with highly variable and extreme climatic conditions (Cohen et al. 2026). Parmesan (1996) was among the first studies to credibly disentangle climate change from other anthropogenic effects in the case of a checkerspot butterfly *Euphydryas editha* in western North America. The species occurs with its host plant in highly local patches, and its conspicuous life history allows for rapid assessment of persistence. Additionally, the butterfly's resource plants and life cycle are finely tuned to the predictable Mediterranean climate (Parmesan 1996). After eliminating land‐use changes as a possible mechanism, Parmesan showed that colonization and extinction patterns in the *E.editha* metapopulation closely fit the latitudinal and elevational range shifts expected due to climate change. This conclusion was matched in a broader study (Parmesan et al. 1999) of European butterflies with a long history of collection records.

Identifying the most important parameters that drive species range shifts thus requires a granular understanding of the factors most relevant to an organism's natural history. Tropical species rely on year‐round resources and often have flexible or extended breeding schedules due to the relative thermal constancy of their environments (Hau 2001; Amaral and Macedo 2003; Moreno‐Palacios et al. 2025). In turn, the physiology of tropical organisms may also have more constrained optimal temperature ranges (Janzen 1967; Ghalambor et al. 2006; McCain 2009; Shah et al. 2017). Since areas at temperate‐tropical boundaries experience greater temperature extremes than the Tropics on an annual basis, poleward range shifts due to climate change are thought to occur in response to a relaxation of minimum temperatures during the coldest part of the year (Crozier 2004; Dangremond et al. 2020; Osland et al. 2021).

### 1.1 The Natural History of the Green Jay in Texas

The green jay ( *Cyanocorax yncas* ) (Figure 1) is a denizen of woodlands and dense brushlands. Prior to its northward expansion, the green jay was found from just north of the Rio Grande at the southernmost tip of Texas (Figure 2) south to Honduras, with another disjunct distribution from Venezuela to Bolivia (Giese and Hall 2024). The green jay avoids open areas with sparse woody cover and tends to travel in dense corridors of vegetation (Gayou 1986), particularly in winter (Oberholser 1974). Nests can be found in a variety of thorny trees and shrubs and generally occur at heights above 2 m (Bendire 1895; Giese and Hall 2024). The open chaparral and savanna to the north of the narrow riparian corridor in the Lower Rio Grande Valley (LRGV) might have been a barrier to northward range expansion in the green jay and other subtropical woodland birds in earlier decades (Brush 2005). The green jay diet is diverse and can include lizards, insects, and the eggs of other bird species (Giese and Hall 2024). During the LRGV winter, when such prey may be periodically unavailable, the jay primarily subsists on Texas ebony ( *Ebenopsis ebano* ) and palm fruits (Gayou 1986). Additionally, as historically widespread LRGV riparian habitat has dwindled in southern Texas, the green jay has come to rely heavily on citrus orchards and suburban yards as food sources and nesting habitats (Brush 2005; Giese and Hall 2024).

**FIGURE 1:**
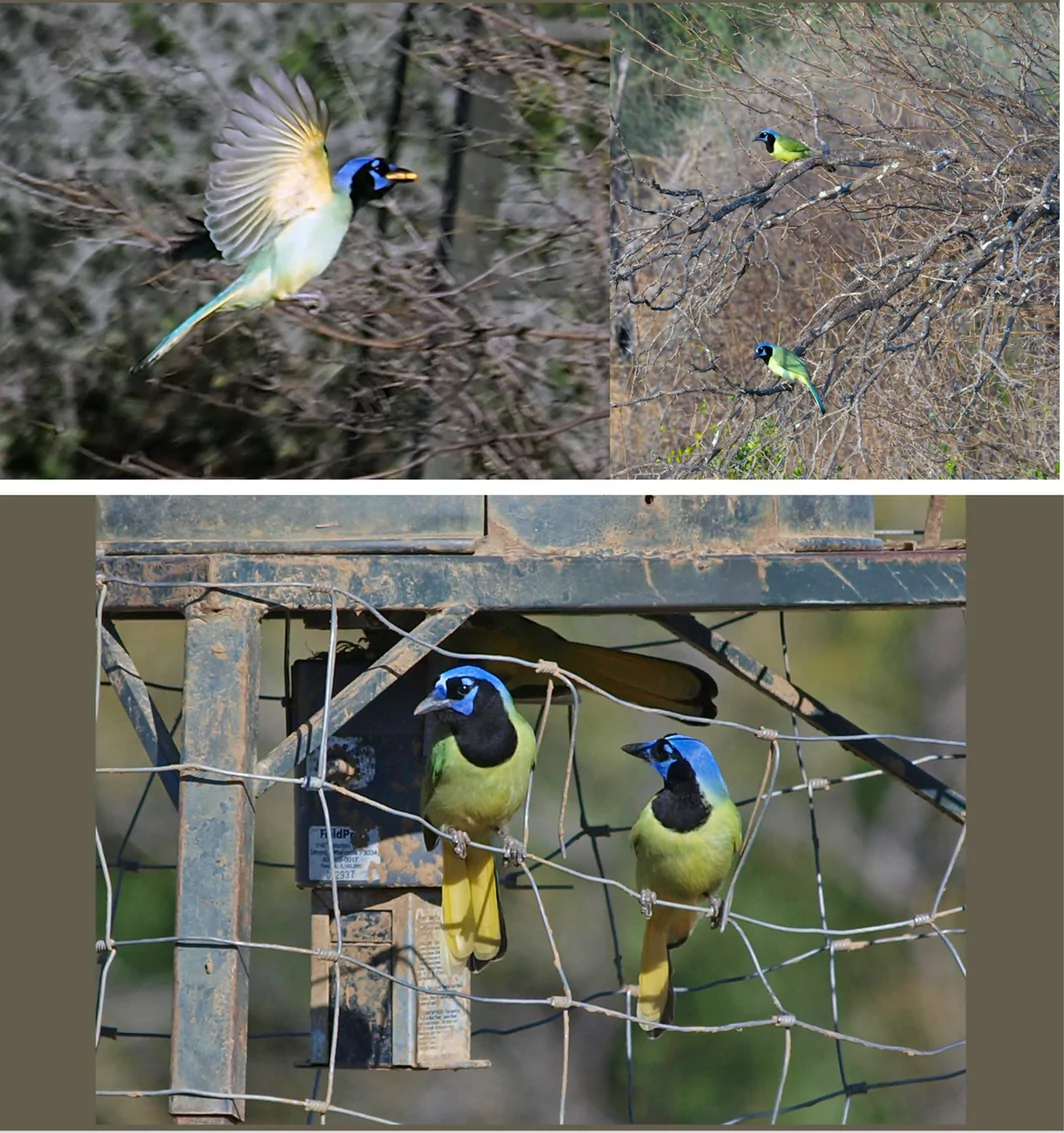
*Top left*: A jay in western Dimmit Co. on a hunting ranch has collected several kernels of corn from ground near a feeder and is headed to a perch. *Top right*: Three green jays muster on an automatic corn feeder in December 2008, on Retama Ranch north of Laredo in Webb County. *Bottom*: Two jays assembled in mesquite tree near an automatic corn feeder waiting for it go off. Photos by L.E.G.

**FIGURE 2:**
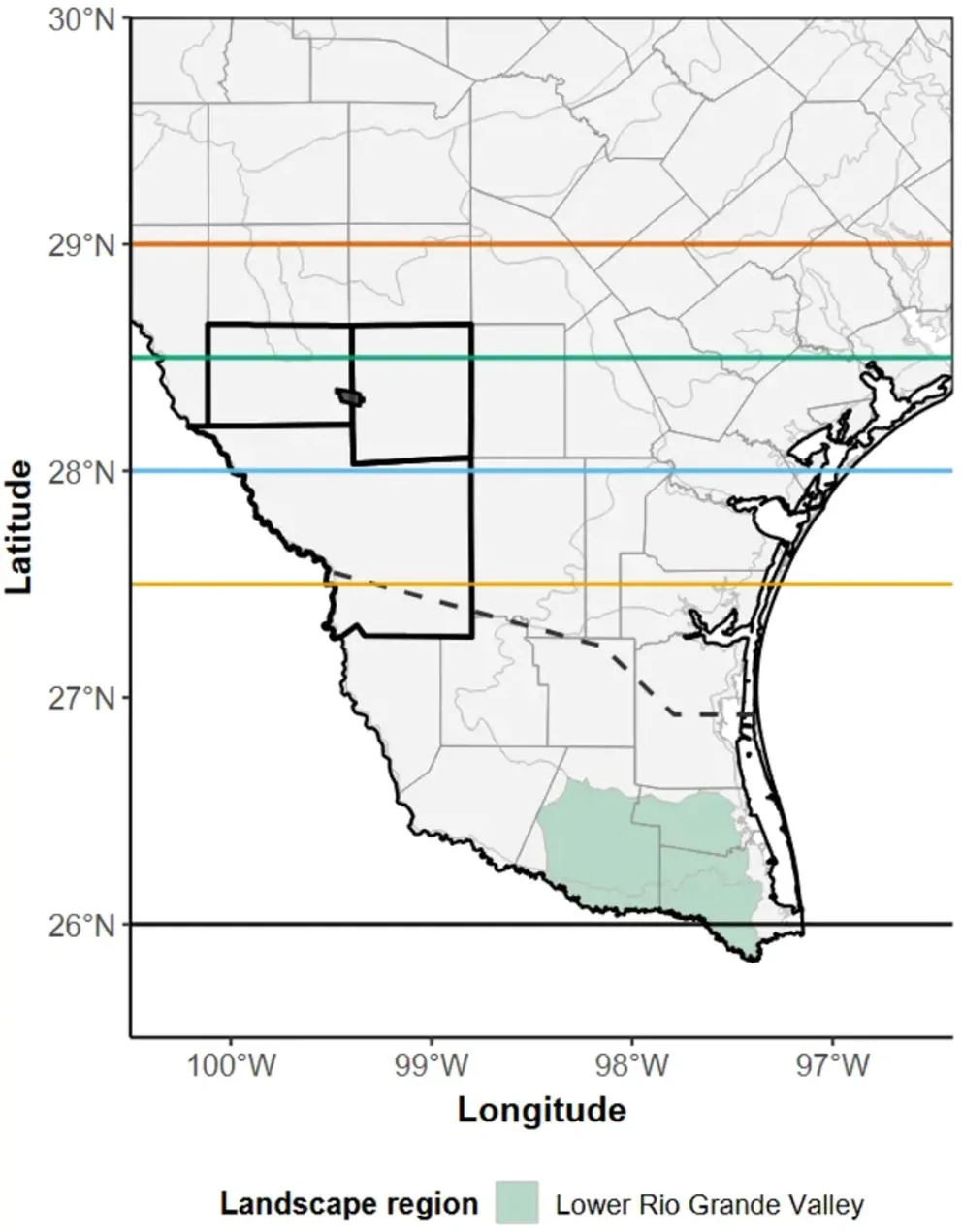
Map providing context about key features of the focal region mentioned in the paper. Colored lines correspond to latitude. 26° N is shown to indicate the southern boundary of the southern Texas region that is the focus of this study. Latitudinal bands from 27.5° N northward show the focal area of the green jay's range expansion since 1970. (A–C) ‘sentinel’ counties (observed by author L.E.G. for 70+ years) from which firsthand accounts are drawn: Dimmit, LaSalle, and Webb Counties, respectively. (D) shows Chaparral Wildlife Management Area, a major source of natural history observations. (E) depicts the rough northern boundary of the green jay distribution in 1970 (Benson and Arnold 2001). Regional freeze exposure was calculated separately across the study region north of 27° N. (F) indicates the Lower Rio Grande Valley ecoregion, the core habitat of the green jay in southern Texas according to observers in 1910 and earlier (Benson and Arnold 2001).

In documenting an intergeneric hybrid between a female green jay and a male blue jay ( *Cyanocitta cristata* ) found near San Antonio, Texas, Stokes and Keitt (2025) hypothesized that the green jay's range expansion of > 2° north may be driven by anthropogenic climate change. To test this hypothesis, it is important to address the regional timing of climate change in relation to the jay's range expansion as well as other anthropogenic changes to habitat. For the green jay to move north, we should expect not only a shift in conditions more favorable to their physiology but also a concordant change in resource availability during the coldest part of the year.

### 1.2 The Climate and Ecology of Southern Texas

The temperate‐tropical transition zone in southern Texas and northeastern Mexico is unique among such gradients globally in that it is exposed on a regular basis to short‐lived continental, polar air masses during the northern winter (Martin 1958; Marengo and Rogers 2001; Kimmel Jr. et al. 2016). For instance, the historic 1951 freeze caused mass die‐offs of deciduous trees throughout the region. During this same polar outbreak, the LRGV experienced 88 consecutive hours of freezing weather (Martin 1958). Similar conditions occurred in the transition zone during January 1963 (Marburger and Thomas 1965) as well as December 1983, December 1989 (Mogil et al. 1984; Lonard and Judd 1991; Bomar 2017), February 2011 (Cruz‐Rico et al. 2015), and February 2021 (Doss‐Gollin et al. 2021). Several of the extreme freezes afflicting southern Texas in the past century immediately followed (1963), preceded (1951), or overlapped (2011, 2021) drought intervals that would have limited resource availability independently of severe cold. Thus, in much of the region to the north of the LRGV, changes in habitat that might have temporarily improved resource availability for a tropical bird like the green jay to the north have often been reversed by extreme drought, cold, or both.

Brief incursions of polar air into the region have historically limited tropical organisms to the Rio Grande corridor or south with only rare occurrences to the north (Vora 1990; Brush 2005). For some tropical insects, like butterfly species staged in northeastern Mexico, periodic northward movement of populations requires not only freedom from extreme cold, but also humid conditions that support adult nectar plants (Gilbert 1969) as well as larval hosts (Cardoso 2010). Among subtropical and tropical birds, a whole suite of species, such as the great kiskadee ( *Pitangus sulphuratus* ), ringed kingfisher ( *Megaceryle torquata* ), white‐tipped dove ( *Leptotila verreauxi* ), and groove‐billed ani ( *Crotophaga sulcirostris* ), have shifted their respective northern range boundaries from approximately 26.5° N to 28° N since 1970 (Rappole et al. 2011). The most parsimonious explanation for parallel, poleward range shifts in ecologically diverse species is global climate change. However, distinguishing climate change from other anthropogenic factors still requires a thorough understanding of an organism's natural history as well as the relative timing of climatic and land use shifts. Over the past 60 years, native habitat in the subtropical southern tip of Texas (LRGV) has increasingly been cleared and converted to suburban and urban development (Brush 2005), and the once‐robust citrus industry has undergone a gradual decline from 40,000 ha under cultivation in the 1940s to 8000 ha in the 2020s (Leffler 1952; USDA 2026) due in large part to episodic droughts and freezes (Sauls and Rouse 1989; Kothari et al. 2026).

In addition to these important land use changes, novel practices such as supplemental corn and protein provided to quail and deer by ranchers have become widespread over the region (Dodd 2009). Additionally, vast but unknown amounts of field corn are added by hunters each fall and winter on lands not formally managed for commercial hunting (reflected in the large piles of “deer corn” outside supermarkets, hardware stores, and service stations across southern Texas each fall and winter). In the 1970s, following the eradication of the New World screwworm ( *Cochliomyia hominivorax* ) in the 1960s (Scruggs 1978), deer hunting on large private ranches in southern Texas began to grow, often accompanied by year‐round supplemental feeding intended to increase hunter access to trophy bucks (e.g., Andre 1991). Energy‐rich corn supplements in the fall and winter hunting season may have allowed for greater survival of green jays north of the LRGV through freezes and droughts, promoting establishment and population growth along the northern fringes of their range. Such resources may facilitate range expansion into new climate regimes, especially if they become available during the part of the year when natural food sources are limited (Valentine et al. 2020).

Here, we reconstruct the northward range expansion of the green jay and compare its progression with long‐term climatic trends as well as historically informed scenarios for changes in resource availability via supplemental feeding. The clearest case for a climate‐driven range expansion is one in which the expansion (1) initiates during a period of warming, (2) progresses in tandem with climate amelioration, and (3) varies on a finer scale according to the frequency or duration of recent severe freezes. Range expansion that began during an unusually cold period would argue against climate change as an initiating mechanism, but later progression could still be facilitated by warming.

Due to the private nature of Texas ranches, quantitative historical data on supplemental feeding are comparatively limited, precluding equivalent statistical tests of its effects. To show that corn‐feeding is a widespread practice across the landscape over which green jay expansion took place, we first map the contemporary spatial distribution of ranches with publicly available documentation of supplemental feeding. We then construct historical scenarios for supplemental feeding in southern Texas based on the timing of technological innovation and important changes to deer populations and wildlife management. We then compare these scenarios with the timing of green jay range expansion and climate change. If increasing food availability contributed to initial colonization, the trajectory of this resource on the landscape should coincide with or precede northward expansion, particularly if expansion began before winter warming. Alternatively, if supplemental feeding increased only after expansion was already underway, it would be unlikely to explain why it initiated. Support for an interaction between food availability and climate would be indicated if food availability preceded or accompanied initial range expansion under otherwise unfavorable climatic conditions, followed by more sustained expansion as severe winters became less frequent. We thus evaluate whether the timing and progression of green jay range expansion are more consistent with climate change acting as an initial driver, food availability facilitating initial colonization, or the two mechanisms acting together.

## 2 Methods

Qualitative information about the green jay's Texas distribution was gathered from early ornithological sources (Smith 1910; Strecker Jr. 1912), the authoritative *The bird life of Texas* (Oberholser 1974), the Texas Breeding Bird Atlas (Benson and Arnold 2001), and firsthand accounts from observers in a handful of sparsely sampled ‘sentinel’ counties (Figure 2). For quantitative analysis in the statistical software R (R Core Team 2026), we aggregated eBird observations (eBird Basic Dataset 2026) into 0.25° × 0.25° spatial cells annually (*N* = 4983 cell‐years) with the package “sf” (Pebesma and Bivand 2023) and modeled Green Jay detections relative to total checklist number using a quasibinomial generalized additive model (GAM) (Wood 2017). The model included a smooth effect of year and a time‐varying effect of latitude, allowing the latitudinal gradient in detection probability to change through time, while controlling for longitude and checklist protocol composition. We used the derivative of the time‐varying latitude effect and its 95% confidence interval to identify periods of sustained northward change. For visualization of spatial changes (Figure 3), observations were aggregated by decade and restricted to adequately sampled cell‐decade combinations. Full data‐processing details, model specification, and justification of the visualization threshold are provided in the Supporting Information S1.

**FIGURE 3:**
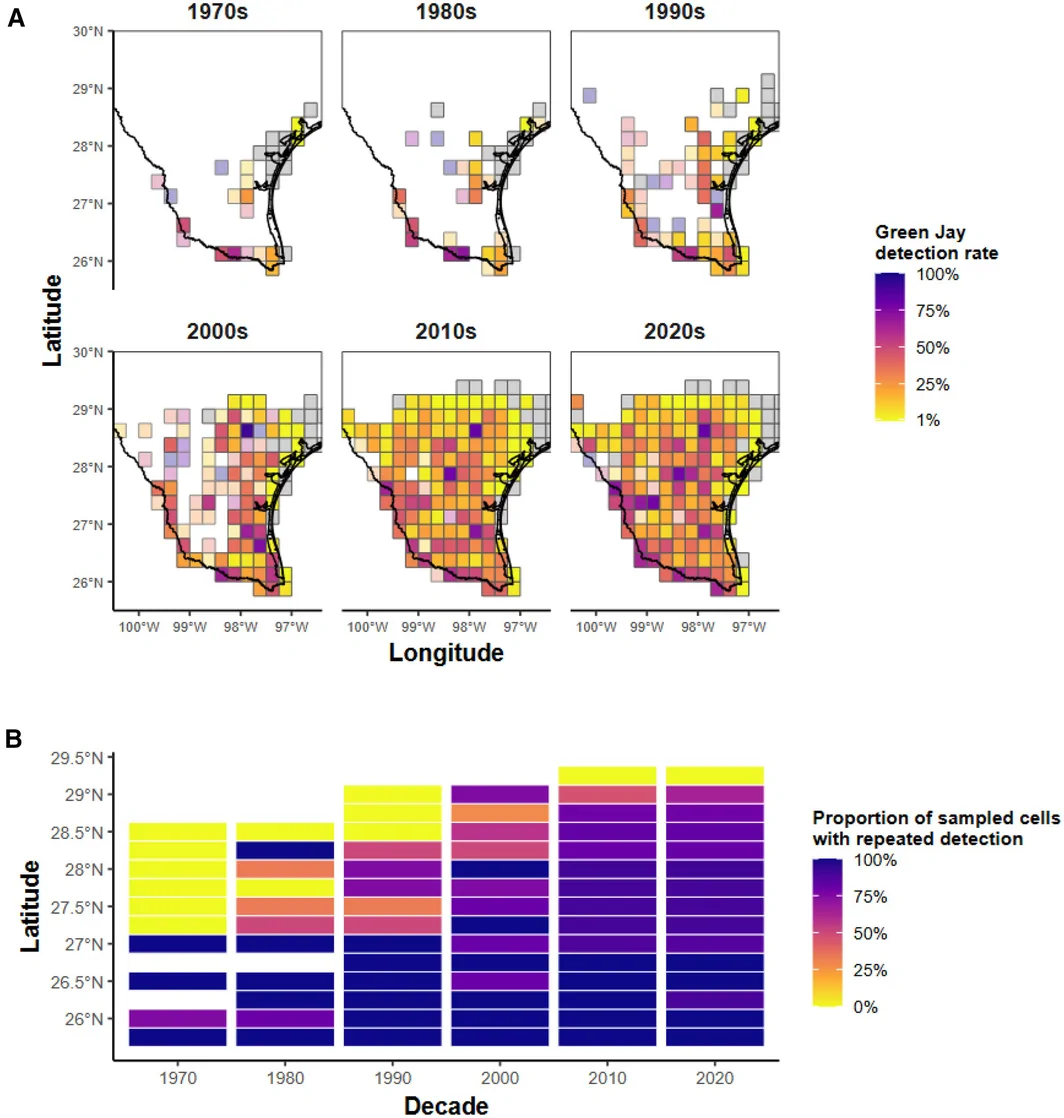
Green jay range expansion in Texas, 1970–2026. (Panel A) shows the gradually increasing detection rate of green jays north of the Lower Rio Grande Valley using 0.25° × 0.25° spatial cells. Each cell is colored according to the proportion of complete eBird checklists reporting a green jay sighting. Full‐color cells met the minimal requirement of at least 20 complete checklists in each decade. Faintly colored cells show green jay detections (on 1 or more checklists) that occurred in cells with fewer than 20 complete checklists. Gray cells had at least 20 complete checklists from that decade but no green jay detections. (Panel B) aggregates adequately sampled cells into 0.25° latitudinal bands. For each latitudinal band, we show the proportion of sampled cells with repeated detection of green jays (present on at least 2 independent checklists during that decade). Latitudinal bands that initially had a high proportion of cells without repeated detections become rarer at higher latitudes as range expansion progresses.

### 2.1 Winter Climate and Severe‐Freeze Exposure

We evaluated winter climate at two temporal resolutions. To place Green Jay expansion in long‐term climatic context, we used PRISM monthly minimum‐temperature estimates from 1931 to 2025 (PRISM 2026). We calculated winter temperature anomalies across four locations spanning the northern portion of the study region near 28° N and modeled their temporal trajectory using a Gaussian GAM. We used the derivative of the fitted smooth to identify periods of detectable warming and ranked all possible 10‐year windows using the R package “slider” (Vaughan 2025) to determine whether expansion began during unusually mild climatic conditions.

To quantify severe winter conditions at a finer temporal scale, we used daily minimum temperatures from NOAA nClimGrid‐Daily for 1951–2026 (Durre et al. 2022). We defined severe freeze days as those with minimum temperature ≤ −2.22°C (28°F), corresponding to a “hard freeze” (NWS 2026), and calculated regional winter freeze exposure at 27° N and north, encompassing the landscape immediately north of the green jay's historical LRGV distribution and across which all subsequent expansion has occurred. We modeled severe‐freeze frequency through time with a Gaussian GAM and used its derivative to identify periods of decline.

We used an event‐history analysis (Landes et al. 2020) to test whether recent severe winters predicted subsequent Green Jay expansion. For each spatial cell, we distinguished first detections from subsequent repeated detections and characterized recent freeze exposure as the maximum regional severe‐freeze frequency during the preceding two winters. We modeled event probability using a binomial GLM including detection stage, freeze exposure, latitude, year, and cell‐year checklist effort, with standard errors clustered by spatial cell. Complete climate‐processing procedures, model specifications, and sensitivity analyses are provided in Supporting Information S1.

### 2.2 Mapping the Spatial Distribution of Supplemental Feeding

We also collected information about the spatial distribution of hunting ranches with evidence of corn feeding across the region from web searches in September 2026. Searches were conducted initially across the region and secondarily on a county‐by‐county basis using combinations of terms related to deer hunting, ranches, supplemental feeding, corn, and feeders. We included properties for which available sources explicitly mentioned the use of corn feeders or corn supplementation during part of the year. For each property, we recorded its reported acreage when available and mapped its location using listed coordinates or the most precise locality information provided by the source. Because many hunting ranches and feeding records are private, these represent a sample of publicly documented ranches that have served as a potential anthropogenic food source for green jays.

### 2.3 Historical Reconstruction of Supplemental Feeding

We reconstructed the historical development and magnitude of supplemental deer feeding in southern Texas using published accounts of deer management, changes in wildlife‐management policy, and economic data. Because annual estimates of supplemental feeding are currently unavailable, we did not model feeding prevalence as a quantitative time series. Instead, we identified historical events and policy changes (Winkler 1983) associated with the encouragement of deer management that allowed supplemental feeding and compared their timing with green jay range expansion and temporal change in winter temperatures. We estimated the magnitude of supplemental corn inputs using data from Dodd (2009), which surveyed 334 ranches encompassing 2.3 million acres across approximately 23 million acres of South Texas. Survey respondents reported expenditures in the year 2006 associated with wildlife management, including supplemental feeding of quail and deer.

Following Dodd (2009), we calculated the acreage receiving deer‐feeding supplements by dividing total supplemental‐feeding expenditures ($1,121,854) by the reported per‐acre feeding cost ($8.41 acre^−1^ year^−1^). Because feeding expenditures included corn, cottonseed, protein supplements, and feeder maintenance, but based on L.E.G.'s personal observations and discussions with hunting ranch owners or managers, corn expenditures can vary from 35% to 100% of the total for supplements added depending on the ranch. Thus, we conservatively assumed that approximately 50% of supplemental‐feeding expenditures for deer were attributable to corn. Corn expenditures were converted to pounds of corn using 2006 grain corn prices ($3.21 bushel^−1^; 56 lb. bushel^−1^). Regional estimates were extrapolated from survey respondents to the broader South Texas region and subsequently scaled to the Nueces Strip, which comprises approximately 61% of the surveyed area. This represents most of the region over which the green jay has expanded since at least the 1960s. Additional details and assumptions underlying extrapolation of feed expenditures to regional corn inputs are provided in the Supporting Information S1.

## 3 Results

Historical records indicate that the green jay began expanding beyond its former distribution exclusive to the Lower Rio Grande Valley by the 1960s (Oberholser 1974; Figure 2). Breeding birds were recorded near 27.5° N along both the Rio Grande and Gulf Coast, although occurrence remained irregular near the northern range margin (Oberholser 1974; Benson and Arnold 2001). Subsequent observations indicate establishment farther north and inland during the 1980s. Consistent with these historical accounts, the eBird analysis detected a strong time‐varying latitudinal effect (edf = 5.53, *F* = 255.41, *p* < 0.001) at both annual (Table S1) and decadal (Table S2), with derivative analysis identifying sustained northward change from approximately 1980–2021 (Figure 3).

### 3.1 Winter Climate During Range Expansion

Green jay expansion did not begin during an unusually mild climatic period. Regional winter temperature showed detectable warming from approximately 1983–1991 (GAM: edf = 3.43, *F* = 3.16, *p* = 0.017; Figure 4C), after northward expansion was already underway (Figure 3). Indeed, 1973–1982 was the coldest of all 86 possible 10‐year windows in the 95‐year record (Figure 4C). Severe‐freeze frequency subsequently declined, with a detectable decrease from approximately 1982–1996 (Figure 4A).

**FIGURE 4:**
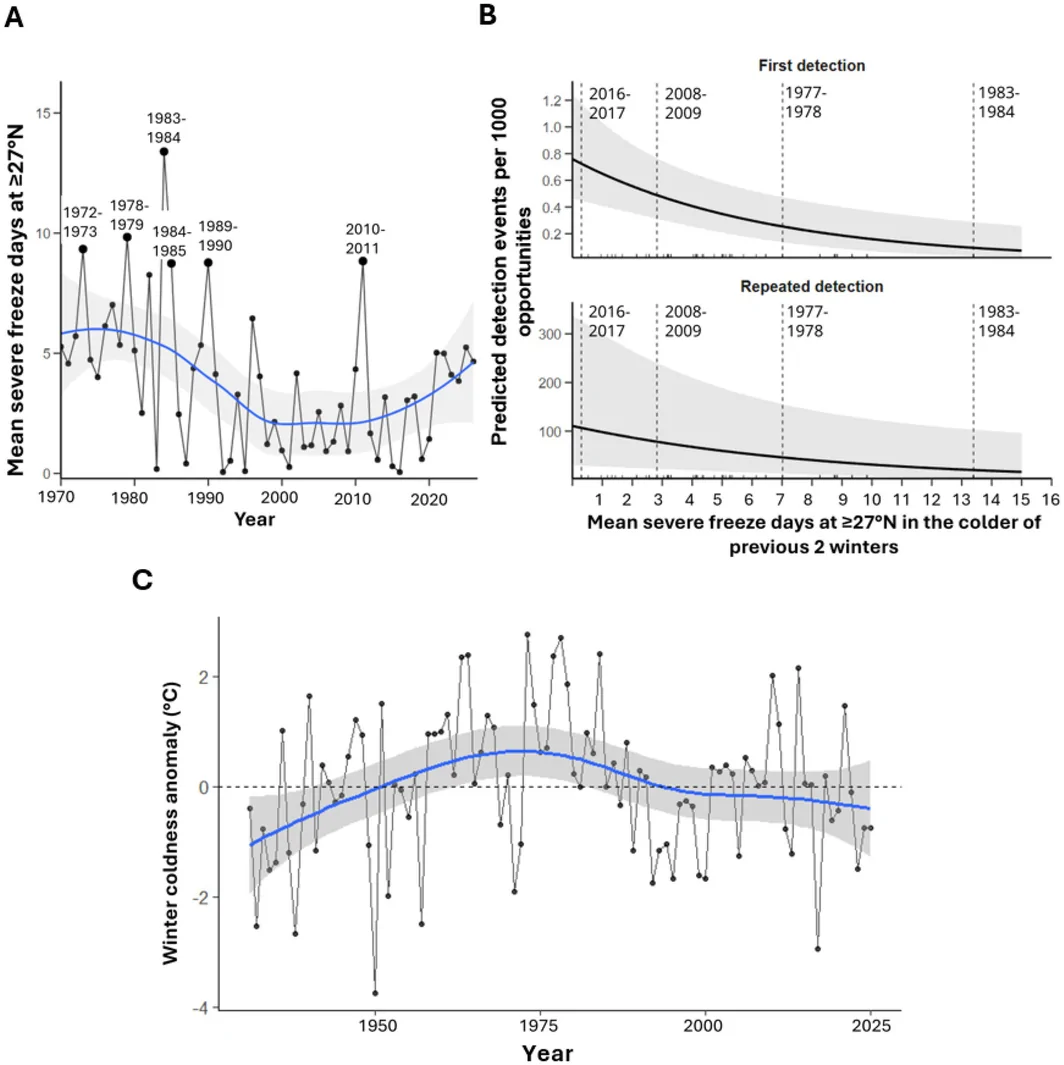
(Panel A) shows a time series of winter severity over the course of the expansion period. The six winters with the highest number of nights below 28°F (−2.22°C) are depicted on the plot. (Panel B) depicts the modeled relationship between winter severity and the rates of first and repeated green jay detections. Lines show predicted numbers of detection events per 1000 potential opportunities for detection. These opportunities are years in which a grid cell had either not yet recorded its first detection (top panel) or, following a first detection, had not yet recorded a repeated detection. Shaded regions represent 95% confidence intervals. Winter severity was quantified as the maximum mean number of severe freeze days during the preceding two winters. The *y*‐axis scale differs between the first‐ and repeated‐detection panels. (Panel C) depicts the regional winter cold anomaly derived from four PRISM grid cells distributed along the 28° N line of latitude, 1931–2025. Temperature anomalies were multiplied by −1 so that positive values represent colder‐than‐average winters and negative values represent warmer‐than‐average winter. Solid line shows the fitted GAM trajectory, and shading indicates the 95% confidence interval.

However, severe winter conditions were negatively associated with subsequent green jay expansion events on a finer scale. In the event‐history model, the probability of a first detection declined with the maximum mean number of severe freeze days during the preceding two winters (*β* = −0.508 ± 0.146 SE, *z* = −3.49, *p* < 0.001; Table S4). We did not find evidence that the effect of freeze exposure on detection probability differed between first and repeated detections (*p* > 0.10). The estimated coefficient for the effect of severe freeze frequency on repeated detections was also negative (*β* = −0.369). We found no evidence that the relationship between severe freeze exposure and expansion probability exhibited a threshold pattern or other form of nonlinearity. When freeze exposure was modeled as a penalized smooth, the estimated relationship was linear (edf = 1.001, *χ* ^2^ = 15.97, *p* < 0.001).

### 3.2 Spatial Distribution of Ranches With Supplemental Feeding

Our searches yielded 113 publicly documented white‐tailed deer supplemental corn‐feeding operations across southern Texas, 112 of which had precise enough location information available for mapping (Figure 5). Corn feeder counts were available for 54 of these properties. However, because corn feeder counts were reported primarily for < 300‐acre properties and for only a handful of the larger ranches (> 5000 acres), we focused on simply mapping the spatial distribution of ranches on the landscape rather than feeder density. The mapped ranches represent a fraction of the total number of supplemental deer‐feeding operations in southern Texas, as the many private ranches and non‐commercial hunting properties throughout the region are not publicly documented.

**FIGURE 5:**
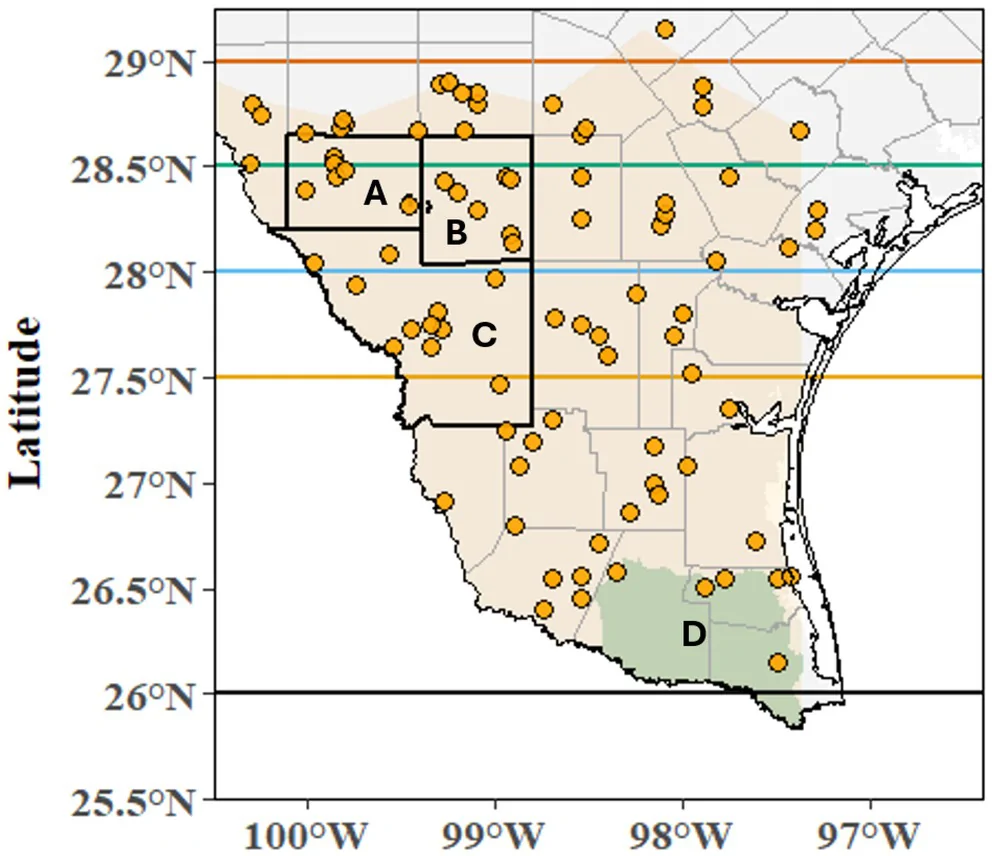
Orange points on the reference map show a sample of publicly reporting white‐tailed deer hunting ranches (*N* = 112) that utilize corn feeders during all or part of the year in management. Latitudinal lines are shown at 0.5° intervals from 27.5° N northward, corresponding to the northern margin of the green jay's 1970 breeding distribution. 26° N is shown to demarcate the southern boundary of the southern Texas region. Bolded outlines of (A–C) denote the ‘sentinel’ counties of Dimmit, La Salle, and Webb, respectively. (D) indicates the Lower Rio Grande Valley ecoregion that was the focal area of the green jay's historic range prior to expansion (Smith 1910; Oberholser 1974). Hunting ranches generally occur to the north and west of this region. On the map, orange dots artificially follow highway corridors due to the official property addresses, but headquarters are often located away from these entrances. A large but unknown fraction across the region carry out similar management practices for family and friend hunting or private lease arrangements. Blank areas on the map reflect large areas of private lands for which public management information is not available. This is the deer corn equivalent of dark matter in space. We refer to it as the “hidden orange matter” (represented as a transparent orange overlay) of southern Texas that, in addition to the publicly advertised properties, may have facilitated northward range expansion of the green jay north of the LRGV.

### 3.3 Historical Development and Magnitude of Supplemental Feeding

By 2006, supplemental feeding represented a widespread anthropogenic resource across southern Texas. Of the 2.3 million acres (930,776 ha) represented in Dodd's (2009) survey of feeding expenditures in 2006, approximately 46% of the area was managed for hunting and roughly 80% of that area was managed specifically for deer hunting. Based on reported feeding expenditures, we calculated that 133,395 acres within the survey's responding ranches received supplemental deer feed. Our extrapolations suggest that corn inputs within the Nueces Strip may have reached tens of millions of pounds annually by 2006. Although we cannot quantify the rate of increase through time, reconstruction of hypothetical supplemental feeding trajectories according to Texas wildlife policy changes and tax law impacting management history suggests that corn availability increases would have been concurrent with green jay range expansion (Figure 6). Additional information is provided in the Supporting Information S1.

**FIGURE 6:**
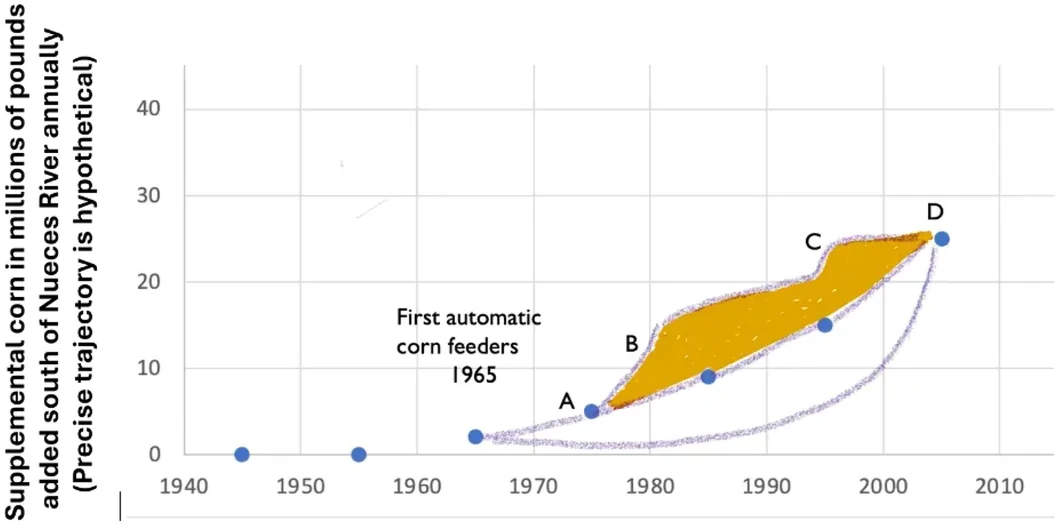
In the 1940s‐1960s, white‐tailed deer were sufficiently rare in southern Texas that only bucks could be harvested and does were off limits. Sterile male New World screw‐worm release started in 1962, leading to eradication in Texas by 1966 (Scruggs 1978). Because white‐tailed does were not hunted and frequently produce twins, populations already had begun increasing dramatically by 1970, soon triggering changes in deer management policies by the Texas Parks and Wildlife Department (TPWD). In 1975, the agency allowed doe harvest for population control (A) and in 1979 (B), further policy shifts enabled commercialized hunting and indirectly increased adoption of supplemental feeding (Winkler 1983). This trend in wildlife management was further amplified by the Texas Legislature's passage of Proposition 11 in 1995 (C), which provided wildlife management comparable tax benefits of agricultural use of private lands. The trend in corn added to Texas south of the Nueces River from near zero in 1960, to a conservative extrapolation of > 20 million pounds in the mid‐2000s (D) from Dodd (2009) can be achieved as an exponential increase (lower line), a roughly linear increase (middle line) or as a series of abrupt steps driven by landowner responses to government policy and regulatory changes (top line). The gold area above the more linear trend line represents a testable hypothesis about the main drivers (A, B) of supplemental feeding.

## 4 Discussion

We investigated the degree to which the timing of the green jay's range expansion north of the Lower Rio Grande Valley (LRGV) coincided with or followed reductions in the severity of extreme winter temperatures. Our quantitative analysis of the more extensive records beginning in 1970 found that the green jay's statistically detectable range expansion (1980–2021) coincided with a reduction in winter freeze severity (1982–1996; Figure 3, Figure 4A). Additionally, on a finer scale, we found that rates of new and repeated detections on the landscape were negatively associated with winter freeze severity, indicating that harsh freezes might have limited successful colonization and persistence of green jays north of the Lower Rio Grande Valley (Figure 4B). Thus, we find support for the idea that climate change during the coldest part of the year may have facilitated northward range expansion of the green jay in Texas (Stokes and Keitt 2025), consistent with parallel range shifts across a suite of bird species in the region (Rappole et al. 2011).

However, while climate change may have facilitated continued range expansion of the green jay, its timing is insufficient to explain why this process initiated. The 1970s to early 1980s contained some of the coldest winters of any decade in the 95‐year climate record, with 1973–1982 being the coldest of all 86 10‐year periods. Yet historical records indicate that northward range expansion was underway by the 1960s–1970s, and eBird records detected sustained northward changes to the distribution beginning around 1980, near the end of an especially cold climate interval. Conversely, several similarly warm periods occurred much earlier: four of the ten warmest 10‐year periods occurred during the 1930s–1950s, decades before northward expansion is evident in the historical record. Thus, warmer winter conditions alone are insufficient to explain the initiation of range expansion, even though finer‐scale analyses show that severe winters might have limited successful colonization.

Green jays were first documented moving north along US Highway 77 in the eastern part of the region after picnic tables and garbage cans were placed beside live oak mottes in the 1960s (Oberholser 1974), suggesting that anthropogenic food resources could have facilitated expansion even during its earliest stages. Supplemental feeding of deer on hunting ranches may subsequently have provided a much more extensive resource. Information gleaned from web searches and decades of personal observations (L.E.G.) suggests that supplemental feed is typically concentrated on intensively managed hunting properties, with automatic feeders spaced approximately 0.5–1.5 km apart (approximately 100–240 ha served by a single feeder). Consequently, supplemental food occurs as discrete resource patches, or “corn islands,” rather than uniformly across the landscape. Assuming green jays utilize space similarly to other social corvids (Gayou 1986; Johnson et al. 2016), individual social groups could potentially access multiple feeders within a single home range. Hunting ranches could therefore function as resource‐rich “stepping stones” providing reliable winter food across a landscape in which suitable habitat and predictable food resources are otherwise patchily distributed.

Our spatial survey strengthens this interpretation by showing that supplemental feeding is widespread across the landscape (Figure 5). The properties shown here are only a small fraction of the total number of supplemental feeding operations in southern Texas and likely underrepresent their extent. The many private operations that do not advertise their supplemental feeding represent a hidden “deer corn dark matter” and would have provided additional support during the green jay's northward range expansion across the same region.

Historical evidence further suggests that the abundance of these anthropogenic food resources increased from the 1970s onward with the aid of management policy changes (figure 6; Winkler 1983). This increase was probably stepwise rather than continuous, producing progressively more opportunities for colonization across southern Texas. Importantly, this trajectory overlaps the period in which the green jay began expanding northward despite cold winter conditions. Thus, we hypothesize that increasing supplemental feeding contributed to a stepwise increase in colonization opportunities, with warmer winter conditions later playing a supporting role.

### 4.1 Potential Consequences of Land Use Changes

In contrast, brush clearing for cattle since the 1950s has not been favorable for northward expansion of the green jay. Chaining, root plowing, and replacement of native grasses with invasive species transformed hundreds of thousands of acres of the Texas brushland savanna, dramatically reducing woody plant diversity (Ruthven III et al. 1993; Figure 7). These anthropogenic changes to the landscape diminished the already sparse woodland habitat of the region at the scale of ranches, which should either limit green jay colonization in the region or require the birds to adopt extensive home ranges at a low density.

**FIGURE 7:**
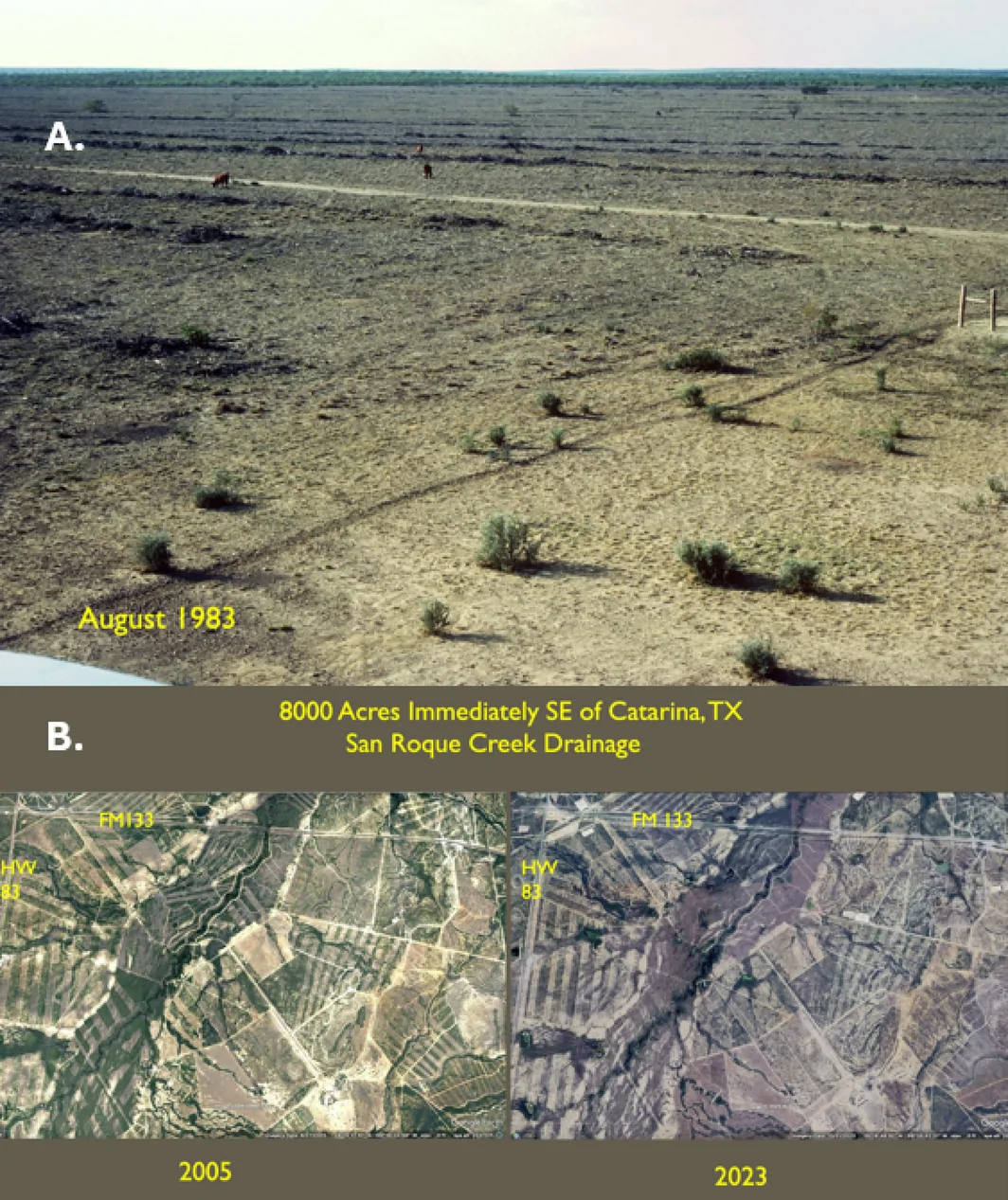
Photographs illustrating landscape‐level anthropogenic changes decreasing habitat suitability for woodland‐dwelling green jays. (A) Root plowed brushland west of Catarina in Dimmit Co. Texas in 1983. This immense ranch has had continuous woody shrub removal from the mid 1950's onward. It continues in 2025. (B) A view of 8000 acres near Catarina, TX. A variety of shrub removal patterns west and east of the main drainage shown immediately southeast of Catarina, defined by intersection of Hwy 83 and FM 133. Much of the San Roque riparian zone was cleared by 2005 and more removed by 2023.

Changes in the LRGV may have promoted northward dispersal or created novel habitat within the green jay's original range. Losses of citrus orchards following severe climatic events, such as extreme freezes and droughts, could have precipitated northward dispersal by the green jay and are noted by Oberholser (1974) as a potential explanation for early sightings north of the LRGV in the 1960s. After repeated losses of citrus in the 1980s, urban, tree‐filled lots spread over former orange orchards and other agricultural fields in the LRGV (Brush 2005; Figure 8). Such residential neighborhoods, which often include irrigated live oaks and bird feeders, provide food and nesting habitat for the green jay. To the extent that the green jay has adapted to urban habitat, this could stage a move to urban settings across southern Texas. Similar habitat changes may have contributed to northward range expansion in other subtropical species associated with suburban habitats, such as buff‐bellied hummingbirds ( *Amazilia yucatanensis* ) and white‐winged doves (Rappole et al. 2011).

**FIGURE 8:**
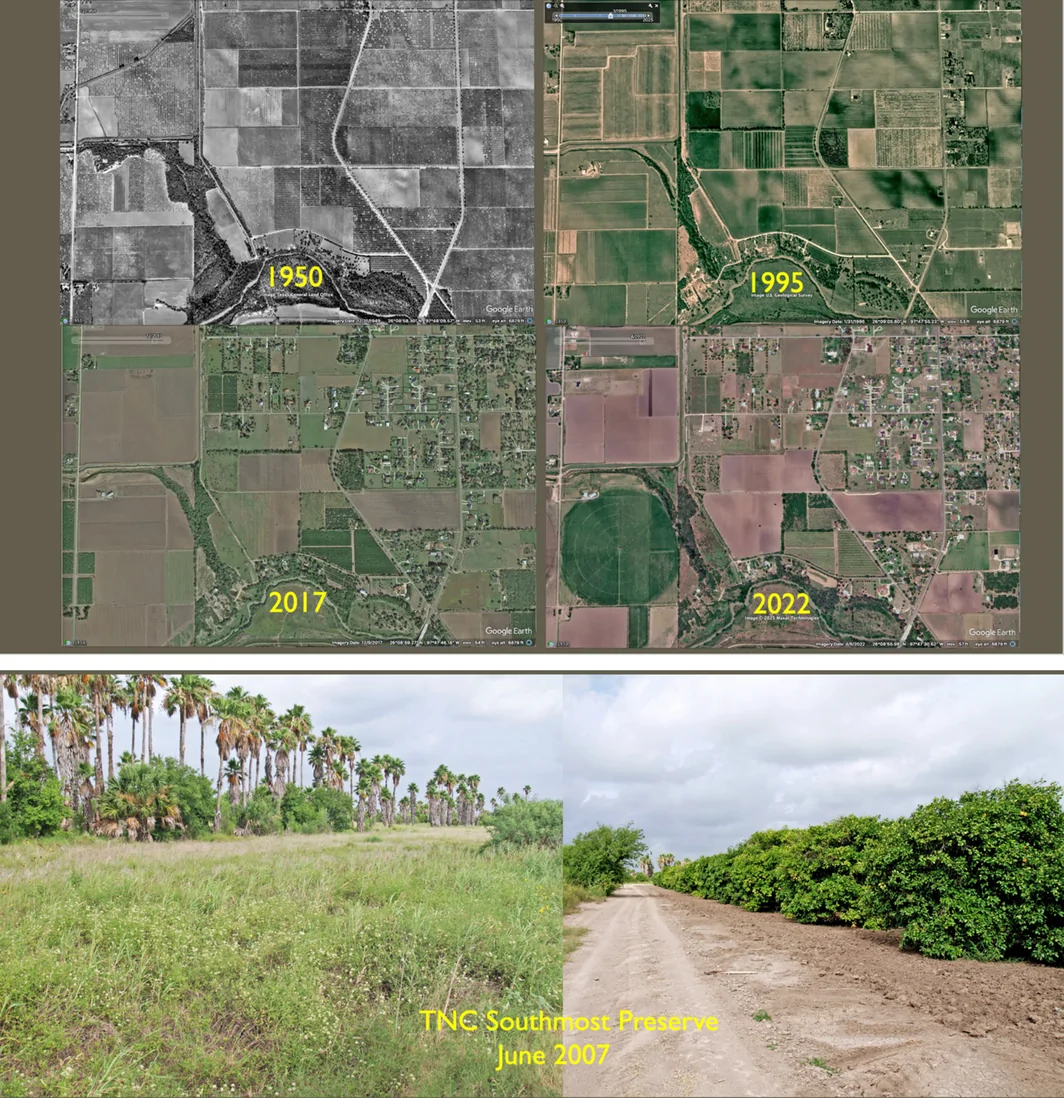
*Top*: An 800‐acre area centered on 26°08′40.87″ N and 97°47′23.03″ W and 5.5 miles SW of Harlingen, TX as shown in Google Earth imagery. In 1950 the site is almost 100% citrus orchards except for native vegetation along Arroyo Colorado. By 1995, losses from freezes in 1951, 1963, 1983, and 1989 progressively reduced return to commercial citrus and, after each climatic extreme, citrus has become a smaller part of agriculture in the site as it has region‐wide (Friend 1995). Additionally, riparian cover has been dramatically reduced along arroyos, and urbanization has begun (Friend 1995; Brush 2005). By 2017, after the 2011 freeze, urbanization has greatly reduced acreage devoted to agriculture and by 2023, after the 2021 freeze, it appears that the little citrus remaining is in recently planted groves, and now urban yards may provide the best food and nesting habitat for green jays. *Bottom*: Southmost preserve of The Nature Conservancy in 2007. Situated on the bank of the Rio Grande in a hairpin bend of the river, this conservation and restoration site is now behind the border wall. The ground level photo by L.E.G. shows the kind of habitat that supported green jays after native subtropical woodlands were converted to citrus and other crops beginning a century ago. These orchards, in place before TNC acquired the property, may not have survived freezes in 2011 and 2021.

Destruction of citrus and other resources in the LRGV following the 1983 and 1989 freezes might have promoted northward dispersal (Figure 4A). While our analyses indicate that severe freezes could have simultaneously reduced the probability of successful colonization and persistence farther north, the consequences of severe freezes during 1983 and 1989 on citrus agriculture in southern Texas were felt for years afterward (Sauls and Rouse 1989; Kothari et al. 2026). Throughout the 1980s and 1990s, dispersing green jays exiting the LRGV would have encountered an increasing number of hunting ranches that provided reliable food, a network of patchy resources that eventually became extensive across southern Texas (Figure 5). Northward dispersal following these freezes was also observed in white‐winged doves (Small et al. 2005). Formerly restricted to the LRGV north of Mexico and now occupying most of Texas, these doves potentially benefited from grain supplements for quail in open pastures of hunting ranches until they reached urban heat islands (Small et al. 2005). Thus, extreme climatic events could simultaneously have acted as a push from damaged habitat and as a constraint on successful establishment, with anthropogenic food subsidies buffering the latter.

## 5 Conclusion

Taken together, our results support a two‐phase hypothesis for the green jay's northward range expansion. Anthropogenic changes in habitat and food availability may have promoted initial colonization along resource‐rich corridors from the 1960s to early 1980s, despite cold winter conditions. Beginning in the 1970s and 1980s, supplemental feeding could have created numerous “corn islands” that allowed dispersing green jays to survive and persist in otherwise marginal habitat. Subsequent climate amelioration might have then relaxed an important constraint on colonization and persistence, potentially allowing expansion to proceed more successfully across southern Texas. However, this occurred concurrently with the spread of a novel anthropogenic resource that would have improved the green jay's ability to survive the climate extremes of the region's northern periphery. Thus, climate change might have only facilitated, rather than initiated, the green jay's northward expansion.

Finally, once established on ranches in our sentinel counties (Dimmit and LaSalle), green jays showed the ability to survive the combined impacts of extreme cold and drought during February 2021 on a ranch in western Dimmit County (L.E.G., personal obs., and O. Longoria, personal comm.). These birds survived exposure to temperatures below 20°F (−7°C) for several days that top‐killed much of the native woody vegetation on the surrounding ranch. It is clear from this case that range shifts of resident organisms in ecoregions like southern Texas, with its high baseline spatial and temporal variation in the constancy, predictability, and contingency of weather extremes (Colwell 1974) have not been adequately addressed by research on organismal responses to climate change. A core question is: to what degree are the key physiological and resource needs of the diverse organisms that move into, within, and through the “intemperate” zone of southern Texas being altered by global warming? The case of the green jay highlights the need to look beyond range boundaries, time, and temperature.

## Author Contributions

**Callen M. Inman:** conceptualization (equal), data curation (lead), formal analysis (lead), investigation (equal), visualization (equal), writing – original draft (lead), writing – review and editing (lead). **Lawrence E. Gilbert:** conceptualization (equal), data curation (supporting), formal analysis (supporting), investigation (equal), visualization (equal), writing – original draft (supporting), writing – review and editing (supporting).

## Funding

The authors have nothing to report.

## Conflicts of Interest

The authors declare no conflicts of interest.

## Supporting information


**Table S1:** Generalized additive model of temporal change in green jay detection probability across latitude, 1970–2026. The quasibinomial GAM modeled the number of green jay detections relative to the total number of complete checklists within 0.25° × 0.25° cell‐year combinations (*N* = 4983). The model included smooth effects of year and the time‐varying effect of latitude, with longitude and checklist protocol composition of each cell‐year included as covariates. Traveling checklists were the reference protocol. Estimated degrees of freedom (edf) 1 indicate nonlinear relationships. Parametric terms are presented in S1a, while smooth terms are presented in S1b.
**Table S2:** Results of quasibinomial generalized linear models examining spatial and temporal variation in green jay detection probability across southern Texas from 1970 to 2026. Simple detection rates are visualized in Figure 3. Detection probability was modeled from the number of green jay detections relative to the total number of complete eBird checklists within each 0.25° × 0.25° cell and decade combination. Models included latitude, longitude, decade, a latitude x decade interaction, and the proportion of checklists belonging to each category. “Traveling” was used as the reference category. Results are shown for the primary analysis that included all sampled cell‐decade combinations.
**Table S3:** Results from a sensitivity analysis using the same models outlined in Table S1, but only using cell‐decade combinations that had adequate sampling (including at least 20 checklists in each decade). In general, findings were not sensitive to the sample size reduction involved in the sensitivity analysis, except for the latitude × decade interaction, which was no longer reached the significance threshold.
**Table S4:** Binomial event‐history model testing associations between severe winter conditions and first and repeated green jay detections north of 27° N. Each observation represents a 0.25° × 0.25° cell and year combination in which a cell was at risk of either its first detection or a subsequent detection. Severe‐freeze exposure was defined as the greater of the regional mean numbers of severe freeze days (daily minimum temperature ≤ −2.22°C) during the two winters preceding each cell‐year combination. Severe freeze exposure, latitude, and year were standardized across the combined first‐ and repeated‐detection data. The number of eBird checklists within each cell‐year was included as a log‐transformed covariate to account for sampling effort. First detection was the reference event type. Standard errors were heteroskedasticity‐consistent (HC1) robust standard errors clustered by spatial cell. Odds ratios for standardized predictors represent the change in odds associated with a one standard‐deviation increase. Odds ratios for interaction terms are the ratios of odds ratios.

## Data Availability

The data are available on Dryad: https://doi.org/10.5061/dryad.80gb5mm5t.
