# Supplementary material for: Corn or Climate? Climate Change Facilitated, but Did Not Initiate, the Northward Range Expansion of the Green Jay in Texas
Source: Ecol Evol. 2026 Oct 18;16(10):e74513. doi: 10.1002/ece3.74513 (PMC13677283; doi:10.1002/ece3.74513)
Supplement: Supplementary file 1 [file ECE3-16-e74513-s001.docx]

**Supplementary Material**

***Supplemental Methods: reconstruction of green jay range expansion***

To quantitatively analyze changes to the green jay’s distribution in Texas, we downloaded all complete eBird checklists recorded between January 1, 1930 and May 31, 2026 from the entire southern Texas region: 25.95°N to 29.20°N and 99.66°W to 95.85°W (Figure 2; eBird Basic Dataset 2026). For purposes of sampling, the region was divided into 108 0.25° x 0.25° latitude x longitude cells. Sampling was sparse and uneven prior to 1970. Between 1930 and 1969, 7,356 checklists reached only 80 (74%) of these spatial cells. By contrast, in the 1970s, 8,453 checklists reached 106 of the 108 cells (98%). We thus only quantitatively analyzed green jay distributional changes from 1970 to 2026, despite evidence of expansion in the 1960s (Oberholser 1974). Ultimately, our analysis contained 804,225 checklists, including 214,820 green jay detections and 589,405 non-detections.

Because checklist effort metadata (such as number of observers, duration, distance traveled) were disproportionately unavailable in earlier decades, requiring these effort metadata would have excluded the majority of historical records. For example, only 6.9% of all checklists submitted from the 1970s contained effort metadata, while this was present in 99.5% of checklists from the 2020s. Therefore, we retained complete checklists irrespective of effort metadata availability and accounted for sampling intensity at the cell level by modeling green jay detections relative to the total number of checklists within each cell and decade. We also included the composition of checklist protocols (e.g. whether the checklist was incidental or taken by a stationary or traveling observer) per cell-decade combination in case this influenced detection probability. Pooling uncommon protocols as “Other,” we calculated the proportion of checklists within each cell and decade represented by Traveling, Stationary, Historical, Incidental, and Area protocols. These were included as covariates in our model, with Traveling omitted as the reference category.

All analyses were performed using the statistical software R (R Core Team 2026). To determine the timing of the green jay’s northward range expansion, we used the R package “sf” (Pebesma and Bivand 2023) to aggregate checklists within 0.25° x 0.25° cell-year combinations (N = 4,983 cell-year observations from 1970 to 2026). We then modeled the number of green jay detections relative to the total number of checklists with a quasibinomial generalized additive model (GAM) using the R package “mgcv” (Wood 2017). Because we did not expect green jay range expansion to proceed at a constant rate across space and time, we included a smooth effect of year and a varying-coefficient smooth that allowed the relationship between latitude and detection probability to change with time. Longitude and the proportions of checklist categories represented by stationary, historical, incidental, area, and other protocols were included as covariates, with traveling checklists used as the reference protocol. We entered the number of checklists within each cell-year as the binomial denominator, with green jay detections within a cell-year as the numerator, accounting for differences in sampling effort among cells and years. To identify periods of sustained northward expansion, we calculated the first derivative of the time-varying latitude effect with respect to year from the fitted GAM, which allowed us to estimate how the latitudinal gradient in green jay detection probability was changing through time. We considered the latitudinal gradient to be undergoing a detectable northward change during periods when the 95% confidence interval of the derivative at each timepoint was entirely greater than zero.

For visualization of spatial changes in green jay occurrence (Figure 3), we aggregated observations by decade. A spatial cell with at least 20 complete eBird checklists was considered to be adequately sampled during a given decade. We evaluated this threshold empirically using checklist detection rates from well-established cell-decades (at least 50 checklists and at least 10 green jay detections). The lower quartile of detection rates among these cell-decades was 0.138. At this detection rate, 20 independent checklists provided a 94.9% probability of detecting green jay presence at least once in an occupied cell (a 5.1% false-negative rate).

*Climate data processing and analysis*

We sought to understand the green jay expansion in the context of climate over the past 100 years. Because daily minimum temperature data are only available at the station level (rather than interpolated over the region) prior to 1951, we obtained monthly minimum temperature data from PRISM (PRISM 2026) for each winter (December-February) from January 1930 until December 2025. These data were taken from a gridded time series estimate for four locations in the northern part of the region roughly along the 28°N latitude line, corresponding to the region that green jays have colonized since 1970. From west to east, we chose one along the Rio Grande drainage (28.3426, -100.2906), one corresponding to Chaparral Wildlife Management Area (28.3794, -99.4985), one near Choke Canyon Reservoir (28.4445, -98.3315), and one near Aransas National Wildlife Refuge along the coast (28.2532, -96.9528). Since data from the first and last winters (1929-1930 and 2025-2026) were incomplete, they were excluded. Thus, our dataset was comprised of 95 winters. Using the R package “mgcv,” we modeled mean winter minimum temperature as a nonlinear function of year with a Gaussian generalized additive model (GAM) including a penalized smooth of year (k = 10) and fitted by restricted maximum likelihood. To characterize temperature variation across the region, we calculated annual winter minimum temperature-anomalies relative to the long term mean at each of the four PRISM grid cells and averaged these anomalies across locations within each winter. To identify periods of winter warming, we calculated the first derivative of the fitted smooth with respect to year using finite differences and obtained standard errors from the model. We considered winter minimum temperature to be undergoing a statistically detectable increase during periods when the pointwise 95% confidence interval around the derivative was entirely greater than zero. For graphical presentation, regional temperature anomalies were multiplied by -1 so that positive values represented colder-than-average winters and negative values represented warmer-than-average winters. While they do not provide the granularity available in daily temperature data, the PRISM data allowed us to identify warming trends on a longer climatic timescale. As supplemental descriptive statistics, we calculated mean regional winter temperatures for every possible 10-year window in the 1931-2025 period using the R package “slider” (Vaughan 2025) and ranked them from coldest to warmest.

We obtained daily temperature data for the years 1951-2026 in southern Texas from NOAA’s nClimGrid-Daily dataset (Durre et al. 2022). nClimGrid-Daily provides daily temperature estimates at a spatial resolution of 1/24° (nominal grid spacing of 4.6 km). We extracted minimum temperature/TMIN estimates for grid cells overlapping the downloaded eBird records (see earlier Methods). We calculated the mean number of days in each winter (November-March) with minimum temperature ≤ −2.22°C (28°F), the mean maximum consecutive number of days at or below the same minimum temperature in each winter, as well as the number of contributing nClimGrid pixels. This threshold corresponds to a ‘hard freeze’ as defined by the U.S. National Weather Service, which typically kills non-dormant subtropical vegetation (US NWS 2026). Hard freezes can determine the transitional zone between temperate and subtropical communities, due to the sensitivity of tropical vegetation to low temperatures (Stevens et al. 2006). The exceptionally prolonged and severe hard freezes in southern Texas in 1983 and 1989 are estimated to have damaged roughly 67% of all native plant species and killed many introduced palms and citrus species (Lonard and Judd 1991) that provide supplemental food for the green jay north of the LRGV riparian corridor. Such conditions, when prolonged, especially severe, or repeated, are expected to limit the availability of small animal prey and reduce citrus fruit production upon which green jays rely during winter.

The two freeze metrics were strongly correlated (Pearson's r = 0.88), so we used total severe-freeze days as our sole measure of winter severity in the subsequent analyses. We then averaged nClimGrid observations across the entire region north of 27°N (Figure 2). We selected 27°N as the southern cutoff to encompass the landscape immediately north of the green jay’s historical LRGV distribution and across which subsequent northward expansion occurred. We first investigated whether the mean number of severe freeze days north of 27°N changed over the course of the study period. We modeled this variable as a smooth function of winter year using a generalized additive model (GAM) with Gaussian errors and restricted maximum likelihood estimation. The smooth was fit with a maximum basis dimension of *k* = 10. We also examined the first derivative of the fitted smooth to characterize periods during which severe freeze frequency was declining.

Next, we used an event-history framework to examine whether recent severe winters differentially predicted green jay detections. We distinguished first detections from repeated detections in a later year following a first detection. A first detection represented the first green jay detection within an eligible cell. A repeated detection occurred when a cell reached its second year of detection following a first detection. This provided evidence of persistent occupation of a cell. To characterize recent exposure to severe winter conditions, we used the maximum of the mean number of severe freeze days across the two complete winters immediately preceding the focal detection. Cell-years represented opportunities for either a first detection or repeated detection, and event type was classified as either “First detection” or “Repeated detection”. We fit a binomial generalized linear model in which event occurrence was predicted by detection stage, severe freeze exposure, and their interaction. The model also included an interaction between detection stage and latitude, year, and the log-transformed number of eBird checklists within each cell-year, with the logit of the probability that a detection event occurred as the response variable. Because individual spatial cells contributed observations across multiple years and detection stages and thus did not contribute independent observations, we calculated heteroskedasticity-consistent standard errors clustered by spatial cell.

Climatic constraints on range expansion could potentially exhibit threshold responses. Thus, we also tested whether the relationship between severe freeze exposure and new detections was nonlinear. We fitted a binomial generalized additive model (GAM) using “mgcv” in which severe freeze exposure was represented by a penalized smooth (k = 5) to allow for moderate nonlinearity while limiting the potential for overfitting. The model included detection type, latitude, year, cell-year checklist effort, and the detection type x latitude interactions as covariates. We estimated the effective degrees of freedom (edf) of the smooth to determine whether the freeze response differed from linearity.**Top of FormBottom of Form**

***Supplemental Methods: Decadal Analysis and Sensitivity Test***

To test whether green jay detections shifted northward through time while accounting for unequal sampling among cells, we modeled the number of green jay detections relative to the total number of checklists within each cell and decade using a quasibinomial generalized linear model. Decade was expressed as the number of decades since 1970, and latitude and longitude were represented by cell centroids. The model included latitude, decade, latitude x decade interaction, longitude, and checklist protocol composition per cell-decade combination, with the response variable being the logit of the probability that a green jay is detected on a checklist within a particular cell during a particular decade. The number of checklists within each cell and decade entered the model as the binomial denominator, allowing cells with greater sampling effort to contribute proportionally more information to estimates of detection probability. A quasibinomial error distribution was used to accommodate overdispersion relative to the binomial distribution. The latitude x decade interaction term provided the primary test of northward range expansion: a positive interaction indicates that temporal increases in green jay detection probability were greater at higher latitudes. As a sensitivity analysis, we repeated the model using only cell-decade combinations represented by at least 20 checklists, corresponding to the adequately sampled cells in Figure 3A.

**Supplemental Methods: Historical reconstruction of corn feeding across southern Texas**

Dodd (2009) surveyed the economic impacts of hunting operations across southern Texas, providing a snapshot for 2006. The survey encompassed a region of approximately 23 million acres, generally corresponding to the region shown in Figure 2. Of 1,421 ranches contacted, 334 responded, collectively owning or leasing 2.3 million acres, or approximately 10% of the southern Texas region. Because these ranchlands are privately owned and surveys were anonymous, the locations of individual properties are unavailable for spatial analysis. However, Dodd (2009) reported expenditures per acre for hunting-related management. Of the 2.3 million acres owned or leased by respondents, 46% (1.05 million acres) were managed for hunting (deer or quail), of which approximately 80% (~840,000 acres) were managed for deer hunting. Assuming that ranchers owning or leasing land were not disproportionately more or less likely to respond to the survey, these ~840,000 acres represent approximately 10% of the total, unknown area managed for deer hunting in the region in 2006.

Supplemental feeding for quail management provisioning sorghum seed (milo), particularly during winter or drought. Although green jays could benefit directly or indirectly from this supplementation, the open habitat associated with quail management does not provide the vegetation structure required by green jays. We therefore focused our estimate on corn provided as supplemental feed for deer. Dodd (2009) reported a per-acre cost (PAC) of supplemental deer feed of $8.41 ($5.48 for landowners + $2.93 for lessees) and total expenditures (TE) on supplemental feeding of $1,121,854. Dividing total expenditures by per-acre costs (TE/PAC) yields an estimated 133,395 acres receiving deer-feeding supplements among survey respondents. Because this expenditure category includes not only corn but also cottonseed, protein, and maintenance of automatic feeders, we assumed that 50% ($4.21 per acre) of annual supplemental-feeding expenditures went toward corn. At the 2006 corn price of $3.21 per bushel, $4.21 would purchase approximately 1.3 bushels; at 56 lb per bushel, this corresponds to approximately 73 lb of corn per acre. Across the estimated 133,395 acres receiving supplemental deer feed, this yields approximately **9.74 million lb of corn added in 2006**.

We then extrapolated this estimate at two scales. First, if the pattern observed among the 334 respondents held across all 1,421 ranches contacted by Dodd (2009), multiplying the respondent estimate by 4.25 yields approximately **41.4 million lb of corn in the year 2006 for** deer management. Second, because the 2.3 million acres represented by respondents comprise approximately 10% of the 23-million-acre southern Texas region, extrapolating the respondent estimate to the entire region yields approximately **97 million lb of corn per year**. These estimates provide different extrapolations of the magnitude of supplemental corn potentially introduced into southern Texas for deer management. Because data on the spatial distribution of supplemental deer feeding are unavailable for our focal region south of the Nueces River, we assumed that private lands managed for hunting were distributed throughout the approximately 23 million acres of southern Texas encompassed by Dodd's (2009) survey. The region between the Nueces River and the Mexican border (the “Nueces Strip”) comprises approximately 14 million acres, or 61% of Dodd's southern Texas region. Assuming that supplemental corn inputs were similarly distributed across this region, applying this proportion to our regional estimates yields approximately **25–59 million lbs of corn annually** within the Nueces Strip.

Supplemental food is unlikely to be distributed uniformly within this region. Instead, it is patchy at two spatial scales. At the landscape scale, properties managed intensively for deer hunting are interspersed among properties managed for quail and those used primarily for agriculture or ranching. Within large hunting ranches, where natural or managed deer densities are commonly approximately one deer per 20–30 acres, supplemental cottonseed, corn delivered through automatic feeders, and sometimes protein feed are provided at intervals of approximately one feeder per 250–600 acres. This corresponds to linear distances between feeders of approximately 0.5–1 mile. Supplemental corn is also concentrated temporally, with much of it provided during fall and winter management in 2006.

***Supplemental Results: Range Expansion Reconstruction***

The first verifiable records of the green jay breeding north of the LRGV (Figure 2) occurred in the early and mid-1960s at 27.5°N near Laredo along the Rio Grande corridor (Webb County, Figure 2; Oberholser 1974, Benson and Arnold 2001, eBird Basic Dataset) and at the same latitude near the coast (Oberholser 1974, Figure 2), but it was still irregular and local along the northern limits of its range in the 1960s (Benson and Arnold 2001). At Chaparral Wildlife Management Area in the sparsely surveyed Dimmit and LaSalle Counties (Figure 2), the biologist Jimmy Rutledge reported that he banded the first breeding pair of green jays in 1985 (Rutledge, J., personal communication). The rancher Bill Maltsberger reported that he first observed green jays on his LaSalle County (Figure 2) ranch at around the same time in the mid-1980s (Maltsberger, W., personal communication). Webb County resident and birdwatcher Elsa Hull moved to San Ygnacio (~27°N along the Rio Grande, Figure 2) in 1986 and reported that green jays became increasingly common, from occasional backyard visitors to abundant local residents, since that time (Hinojosa, A., personal communication).

Analysis at an annual resolution indicated that the latitudinal distribution of green jay detections changed nonlinearly through time. We found an effect of latitude that varied through time (edf = 5.53, F = 255.41, p < 0.001; Table S1), and an overall smooth effect of year (edf = 5.84, F = 9.79, p < 0.001). Derivative analysis showed a sustained period of detectable northward range expansion from approximately 1980 to 2021.

***Supplemental Results: Decadal Analysis and Sensitivity Test***

Green jay detection shifted northward from the 1970s through the 2020s (Figure 3). After accounting for longitude, sampling protocol composition, and variation in the number of checklists contributing to each cell-decade detection rate, the temporal change in detection probability differed significantly with latitude (latitude x decade: β = 0.155 ± 0.072 SE, *t* = 2.14, *P* = 0.032; Table S2). The positive interaction indicated that detection probability increased more strongly through time at higher latitudes, consistent with northward range expansion. Detection probability also increased through time overall (β = 0.537 ± 0.141, *t* = 3.82, *P* < 0.001) and declined with latitude at the beginning of the modeled period (β = −1.427 ± 0.338, *t* = −4.22, *P* < 0.001). Detection also varied strongly with longitude (β = −1.380 ± 0.063, *t* = −21.87, *P* < 0.001).

Restricting the analysis to cell-decade combinations represented by ≥20 checklists produced a nearly identical estimate of the latitude × decade interaction (β = 0.161 ± 0.099 SE; Table S3), although the reduced dataset resulted in greater uncertainty and the interaction was not statistically significant (*t* = 1.62, *P* = 0.105). Thus, exclusion of sparsely sampled cells did not reduce the estimated magnitude of the northward temporal shift.

*Long-term winter temperature trends*

Regional winter minimum temperature varied nonlinearly throughout the 1931-2025 PRISM record (GAM: edf = 3.43, F = 3.16, p = 0.017; Figure 4C). This winter warming followed a period of exceptionally cold winters: 1973-1982 was the coldest of all 86 possible 10-year windows in the 95-year record (mean winter minimum temperature = 5.60°C), and eight of the ten coldest windows overlapped the 1970s and early 1980s. The warmest conditions occurred later, with 1991-2000 and 1992-2001 representing the warmest 10-year windows (7.94°C and 7.92°C, respectively). However, comparably warm conditions also occurred earlier in the record. The 1932-1941 and 1931-1940 periods were the third- and fourth warmest 10-year windows (7.86°C and 7.78°C, respectively), while 1948–1957 and 1949–1958 ranked eighth and ninth (7.69°C in both cases).

*Severe freeze frequency*

The frequency of severe freezes north of 27°N changed nonlinearly over the 75 winters from 1952-2026 (GAM: edf = 3.93, F = 5.54, p < 0.001; 30.3% deviance explained). The fitted relationship indicated that the decline was primarily concentrated during the 1980s and 1990s. Examination of the derivative of the fitted smooth showed that the clearest declining interval was approximately 1982-1996.

*The relationship between severe freeze frequency and green jay detection*

Severe winter conditions were negatively associated with subsequent green jay expansion events. In the event-history model, the probability of a first detection declined with the maximum mean number of severe freeze days during the preceding two winters (β = −0.508 ± 0.146 SE, z = -3.49, p < 0.001; Table S4). From this model, we computed a 40% reduction in the odds of first detection for each standard-deviation increase in severe-freeze exposure (Odds Ratio = 0.60). We did not find evidence that the effect of freeze exposure on detection probability differed between first and repeated detections (freeze exposure x detection type: β = 0.139 ± 0.254, z = 0.55, p = 0.583). The estimated coefficient for the effect of severe freeze frequency on repeated detections was also negative (β = -0.369). First-detection probability declined with increasing latitude (β = −0.469 ± 0.145, z = −3.22, p = 0.001). We did not find evidence that the latitude effect differed between first and repeated detections (β = 0.390 ± 0.204, z = 1.91, p = 0.056). After accounting for freeze exposure, latitude, detection type, and sampling intensity, there was no residual effect of year (β = 0.192 ± 0.131, z = 1.47, p = 0.141). Checklist effort within each cell-year combination was included as ln(1 + number of checklists) and was not show evidence for an association with event probability (β = −0.132 ± 0.074, z = −1.79, p = 0.074). We found no evidence that the relationship between severe freeze exposure and expansion probability exhibited a threshold pattern or other form of nonlinearity. When freeze exposure was modeled as a penalized smooth, the estimated relationship was linear (edf = 1.001, χ² = 15.97, p < 0.001).

**Estimated corn subsidies across southern Texas**

Based on reported feeding expenditures from the year 2006, we estimated that 133,395 acres within the survey received supplemental deer feed. Assuming half of supplemental-feeding expenditures were allocated to corn, approximately 9.74 million pounds of corn were distributed annually across surveyed properties that chose to respond in 2006. Assuming non-responding ranches averaged similar values to those responding**,** we extrapolated these values to all ranches contacted by Dodd (2009). This yielded an estimate of 41.4 million pounds of deer corn annually. Scaling this estimate to the entirety of South Texas suggested annual inputs approaching 97 million pounds of corn.

With no data available on the spatial distribution of supplemental feeding of deer in our focal area of south Texas, i.e., south of the Nueces River, we can only assume that private lands managed for hunting are scattered across the entire 23 million southern Texas acres sampled by Dodd’s (2009) survey. The region between the Nueces and Mexican Border is about 14 million acres or 61% of Dodd’s “South Texas”. Assuming corn supplement amounts are uniformly distributed over southern Texas, the “Nueces Strip” would have had 25-59 million pounds of corn added for deer management. Such corn resources available to Green Jay are available primarily in fall and winter during rut, and also patchy at two spatial scales. At a large scale, properties that manage intensively for deer hunting are scattered among those that manage for quail or that practice only agriculture/ranching.

At a finer scale within the larger hunting ranches common in this zone with low natural or managed deer densities of 1/20-30 acres, supplements of cottonseed, corn (automatic feeders), and sometimes protein feeders are provided every 250-600 acres. The linear distance between feeders at such density range between .5 and 1 mile. Little is known about home range in Green Jay but social groups likely move between several feeders Telemetry studies of Pinyon Jay in New in New Mexico (Johnson et al. 2016) reveal flock home ranges of up to 15,000 acres. This would be a small to medium-sized hunting ranch in the western edge of the Nueces Strip. Assuming one corn feeder per 600 acres, there would be about 25 automatic feeders within the home range of Green Jays should they forage as widely as their cousin. One can imagine such ranches as islands of predictable resources in a sea lacking needed habitat structure and predictable food. Any Green Jays dispersing northward away from freeze devastated habitat in the LRGV in winter would not likely find food unless they encountered an “island” where corn supplements are provided during winter months by humans.

If this scenario is correct, then trends through time in increase of supplemental feeding for deer management in the Nueces Strip should be a major factor in the spread of Green Jay. Between 1965, when automatic feeders were first employed on a few ranches to 2006 when the input of supplemental feeding of deer can be quantified (Dodd 2009), there were 4 decades of increase in the addition of fall-winter food useful to Green Jay. Two turning points were in 1975, when range conditions and supplemental feeding led to requests for increase in permits to control deer numbers by increasing numbers of antlerless deer permits and then in 1979, when a more formal program of TPWD-approved management plans involving population control (Winkler 1983). These policy shifts correspond to increases in high fences that allowed landowners to better control deer herd numbers, essentially semi-privatizing this wild species and incentivized investing in supplemental feeding as a way to increase the quality and economic value of the trophy male deer available to hunters. We have mentioned the 1995 legislation (Prop 11) allowing wildlife management to count as agricultural use exemption for 90% property tax reduction. This would have further incentivized owners to manage for deer and invest in related costs including supplemental feed. Thus, the trend of increasing corn supplements in S Texas between 1965 and 2005 was likely punctuated and stepwise rather than linear or exponential. We can identify the types of economic data that would bolster observations with numbers:

1. annual sales of automatic feeders,

2. fall and winter grain corn sales in S Texas,

3. increase in high fencing by year encouraged by numbers of wildlife management permits (after 1979) and

4. increase in number of properties in the zone receiving tax reductions for wildlife management Proposition 11 (after 1995)

However, in the absence of continuous numbers, we can predict that TPDW policy shifts in 1975, 1979, as well as State Proposition 11 in 1995, discussed above, each would have abruptly increased the target size of “corn islands” and green jay success in colonizing northwards after severe freezes in the LRGV.

**Table S1.** Generalized additive model of temporal change in green jay detection probability across latitude, 1970-2026. The quasibinomial GAM modeled the number of green jay detections relative to the total number of complete checklists within 0.25° x 0.25° cell-year combinations (N = 4983). The model included smooth effects of year and the time-varying effect of latitude, with longitude and checklist protocol composition of each cell-year included as covariates. Traveling checklists were the reference protocol. Estimated degrees of freedom (edf) 1 indicate nonlinear relationships. Parametric terms are presented in S1a, while smooth terms are presented in S1b.

**a.**

| **Predictor** | **β** | **SE** | **t** | **p-value** |
| --- | --- | --- | --- | --- |
| Longitude | −1.284 | 0.023 | −56.463 | <0.001 |
| Stationary | −0.768 | 0.146 | −5.246 | <0.001 |
| Historical | 0.790 | 0.345 | 2.291 | 0.022 |
| Incidental | 1.451 | 0.231 | 6.276 | <0.001 |
| Area | −0.741 | 0.636 | -1.165 | 0.244 |
| Other protocol | 1.993 | 1.423 | 1.401 | 0.161 |

**b.**

| **Smooth term** | **edf** | **Ref. df** | **F** | **p-value** |
| --- | --- | --- | --- | --- |
| Year | 5.837 | 6.703 | 9.789 | <0.001 |
| Year x latitude | 5.532 | 6.361 | 255..412 | <0.001 |

**Table S2.** Results of quasibinomial generalized linear models examining spatial and temporal variation in green jay detection probability across southern Texas from 1970-2026. Simple detection rates are visualized in Figure 3. Detection probability was modeled from the number of green jay detections relative to the total number of complete eBird checklists within each 0.25° x 0.25° cell and decade combination. Models included latitude, longitude, decade, a latitude x decade interaction, and the proportion of checklists belonging to each category. ‘Traveling’ was used as the reference category. Results are shown for the primary analysis that included all sampled cell-decade combinations.

| **Predictor** | **β** | **SE** | **t** | **p-value** |
| --- | --- | --- | --- | --- |
| Latitude | −1.427 | 0.338 | −4.217 | <0.001 |
| Decade | 0.537 | 0.141 | 3.816 | <0.001 |
| Longitude | −1.380 | 0.063 | −21.873 | <0.001 |
| Stationary protocol proportion | −0.349 | 0.481 | −0.725 | 0.469 |
| Historical protocol proportion | 0.179 | 0.679 | 0.264 | 0.792 |
| Incidental protocol proportion | 3.320 | 0.706 | 4.705 | <0.001 |
| Area protocol proportion | −2.579 | 2.916 | −0.885 | 0.377 |
| Other protocol proportion | 3.939 | 4.754 | 0.829 | 0.408 |
| Latitude × decade | 0.155 | 0.072 | 2.144 | 0.032 |

**Table S3.** Results from a sensitivity analysis using the same models outlined in Table S1, but only using cell-decade combinations that had adequate sampling (including at least 20 checklists in each decade). In general, findings were not sensitive to the sample size reduction involved in the sensitivity analysis, except for the latitude x decade interaction, which was no longer reached the significance threshold.

| **Predictor** | **Β** | **SE** | **t** | **p-value** |
| --- | --- | --- | --- | --- |
| Latitude | −4.021 | 0.895 | −4.491 | <0.001 |
| Decade | 0.526 | 0.181 | 2.902 | 0.004 |
| Longitude | −1.298 | 0.077 | −16.878 | <0.001 |
| Stationary protocol proportion | −0.349 | 0.626 | −0.558 | 0.577 |
| Historical protocol proportion | 0.194 | 0.904 | 0.214 | 0.831 |
| Incidental protocol proportion | 3.438 | 0.930 | 3.698 | <0.001 |
| Area protocol proportion | −3.362 | 4.118 | −0.816 | 0.415 |
| Other protocol proportion | 3.954 | 6.154 | 0.643 | 0.521 |
| Latitude × decade | 0.161 | 0.099 | 1.624 | 0.105 |

**Table S4.** Binomial event-history model testing associations between severe winter conditions and first and repeated green jay detections north of 27°N. Each observation represents a 0.25 x 0.25° cell and year combination in which a cell was at risk of either its first detection or a subsequent detection. Severe-freeze exposure was defined as the greater of the regional mean numbers of severe freeze days (daily minimum temperature ≤ −2.22°C) during the two winters preceding each cell-year combination. Severe freeze exposure, latitude, and year were standardized across the combined first- and repeated-detection data. The number of eBird checklists within each cell-year was included as a log-transformed covariate to account for sampling effort. First detection was the reference event type. Standard errors were heteroskedasticity-consistent (HC1) robust standard errors clustered by spatial cell. Odds ratios for standardized predictors represent the change in odds associated with a one standard-deviation increase. Odds ratios for interaction terms are the ratios of odds ratios.

| **Predictor** | **β** | **Robust SE** | **z** | **p-value** | **Odds Ratio** | **95% CI for OR** |
| --- | --- | --- | --- | --- | --- | --- |
| Intercept | −2.444 | 0.195 | −12.523 | <0.001 | 0.087 | 0.059–0.127 |
| Repeated detection | 1.735 | 0.271 | 6.407 | <0.001 | 5.667 | 3.333–9.634 |
| Severe-freeze exposure | −0.508 | 0.146 | −3.487 | <0.001 | 0.602 | 0.452–0.801 |
| Latitude | −0.469 | 0.145 | −3.221 | 0.001 | 0.626 | 0.471–0.832 |
| Year | 0.192 | 0.131 | 1.473 | 0.141 | 1.212 | 0.938–1.565 |
| ln(1 + cell-year checklists) | −0.132 | 0.074 | −1.786 | 0.074 | 0.876 | 0.758–1.013 |
| Repeated detection × severe-freeze exposure | 0.139 | 0.254 | 0.549 | 0.583 | 1.150 | 0.699–1.891 |
| Repeated detection × latitude | 0.390 | 0.204 | 1.907 | 0.056 | 1.477 | 0.989–2.204 |
